# Copy number variation analysis based on AluScan sequences

**DOI:** 10.1186/s13336-014-0015-z

**Published:** 2014-12-05

**Authors:** Jian-Feng Yang, Xiao-Fan Ding, Lei Chen, Wai-Kin Mat, Michelle Zhi Xu, Jin-Fei Chen, Jian-Min Wang, Lin Xu, Wai-Sang Poon, Ava Kwong, Gilberto Ka-Kit Leung, Tze-Ching Tan, Chi-Hung Yu, Yue-Bin Ke, Xin-Yun Xu, Xiao-Yan Ke, Ronald CW Ma, Juliana CN Chan, Wei-Qing Wan, Li-Wei Zhang, Yogesh Kumar, Shui-Ying Tsang, Shao Li, Hong-Yang Wang, Hong Xue

**Affiliations:** Division of Life Science and Applied Genomics Centre, Hong Kong University of Science and Technology, Clear Water Bay, Hong Kong, China; National Center for Liver Cancer Research and Eastern Hepatobiliary Surgery Hospital, 225 Changhai Road, Shanghai, 200438 China; Department of Oncology, Nanjing First Hospital, No. 68 Changle Road, Nanjing, 210006 China; Department of Hematology, Changhai Hospital, Second Military Medical University, 174 Changhai Road, Shanghai, 200433 China; Department of Thoracic Surgery, Jiangsu Key Laboratory of Molecular and Translational Cancer Research, Nanjing Medical University Affiliated Cancer Hospital, Cancer Institute of Jiangsu Province, Baiziting 42, Nanjing, 210009 China; Division of Neurosurgery, Department of Surgery, Prince of Wales Hospital, Chinese University of Hong Kong, 30-32 Ngan Shing Street, Sha Tin, Hong Kong, China; Division of Neurosurgery, Department of Surgery, Li Ka Shing Faculty of Medicine, University of Hong Kong, Queen Mary Hospital, 102 Pokfulam Road, Hong Kong, China; Department of Neurosurgery, Queen Elizabeth Hospital, 30 Gascoigne Road, Kowloon, Hong Kong, China; Shenzhen Center for Disease Control and Prevention, No 8 Longyuan Road, Nanshan district, Shenzhen City, 518055 China; Nanjing Brain Hospital and Nanjing Institute of Neuropsychiatry, Nanjing Medical University, Nanjing, 210029 China; Department of Medicine and Therapeutics, 9th floor, Clinical Sciences Building, The Prince of Wales Hospital, Shatin, Hong Kong; Department of Neurosurgery, Beijing Tiantan Hospital, 6 Tiantan Xili, Dongcheng District, Capital Medical University, Beijing, 100050 China; MOE Key Laboratory of Bioinformatics and Bioinformatics Division, TNLIST, Department of Automation, Tsinghua University, Beijing, 100084 China; International Cooperation Laboratory on Signal Transduction, Eastern Hepatobiliary Surgery Hospital, 225 Changhai Road, Shanghai, 200438 China

**Keywords:** AluScan sequencing, CNV calling, Cancer classification, Machine learning

## Abstract

**Background:**

AluScan combines inter-*Alu* PCR using multiple *Alu*-based primers with opposite orientations and next-generation sequencing to capture a huge number of *Alu*-proximal genomic sequences for investigation. Its requirement of only sub-microgram quantities of DNA facilitates the examination of large numbers of samples. However, the special features of AluScan data rendered difficult the calling of copy number variation (CNV) directly using the calling algorithms designed for whole genome sequencing (WGS) or exome sequencing.

**Results:**

In this study, an AluScanCNV package has been assembled for efficient CNV calling from AluScan sequencing data employing a Geary-Hinkley transformation (GHT) of read-depth ratios between either paired test-control samples, or between test samples and a reference template constructed from reference samples, to call the localized CNVs, followed by use of a GISTIC-like algorithm to identify recurrent CNVs and circular binary segmentation (CBS) to reveal large extended CNVs. To evaluate the utility of CNVs called from AluScan data, the AluScans from 23 non-cancer and 38 cancer genomes were analyzed in this study. The glioma samples analyzed yielded the familiar extended copy-number losses on chromosomes 1p and 9. Also, the recurrent somatic CNVs identified from liver cancer samples were similar to those reported for liver cancer WGS with respect to a striking enrichment of copy-number gains in chromosomes 1q and 8q. When localized or recurrent CNV-features capable of distinguishing between liver and non-liver cancer samples were selected by correlation-based machine learning, a highly accurate separation of the liver and non-liver cancer classes was attained.

**Conclusions:**

The results obtained from non-cancer and cancerous tissues indicated that the AluScanCNV package can be employed to call localized, recurrent and extended CNVs from AluScan sequences. Moreover, both the localized and recurrent CNVs identified by this method could be subjected to machine-learning selection to yield distinguishing CNV-features that were capable of separating between liver cancers and other types of cancers. Since the method is applicable to any human DNA sample with or without the availability of a paired control, it can also be employed to analyze the constitutional CNVs of individuals.

**Electronic supplementary material:**

The online version of this article (doi:10.1186/s13336-014-0015-z) contains supplementary material, which is available to authorized users.

## Introduction

The use of microarray platforms to perform copy number variation (CNV) calling is a valuable technique in genomic analysis. However, next-generation sequencing is fast becoming an attractive alternative platform for this purpose. Compared to microarrays, next-generation sequencing can make possible a higher resolution, multiple simultaneous analyses on the same sample, and at least comparable detection efficiency in CNV calling [1]. Moreover, while CNV calling from microarrays requires the establishment of a relationship between copy number and the observed intensity for any site-specific probe [2], the read-depth of any fragment in an output of next-generation sequencing can be correlated to the copy number either linearly or based on a simple Poisson model [3,4].

A variety of algorithms have been designed for CNV calling from sequencing data obtained for both paired and unpaired samples [3-10]. In general, data from whole genome sequencing (WGS) are continuous and more evenly distributed so that they are readily fitted to simple statistical distributions following straightforward GC-normalization. On the other hand, CNV calling based on target-capture sequencing such as exome sequencing and AluScan [11], is more complex. As a method for genome-wide capture of the sequences amplified by inter-*Alu* PCR using multiple *Alu*-based primers with opposite ‘head type’ and ‘tail type’ orientations for next-generation sequencing, AluScan is not only expeditious in both experimental and informatics analysis, but also requires less DNA compared to WGS or exome sequencing. However, the sequences analyzed by both exome sequencing and AluScan are discontinuous. Moreover, while exome sequencing usually involves basically the same set of fixed target regions in every experiment, such that CNV calling on an unpaired sample can be performed without any control [7], the inter-*Alu* sequences analyzed by AluScan depend on the *Alu*-based PCR primers employed. As a result, CNV-calling algorithms developed for WGS or exome sequencing are not readily applicable to AluScans. Moreover, it is possible that *Alu* sequences could be one of the factors that induce CNVs, because the high similarity of neighboring *Alu* elements could cause homologous recombination that may result in changes in copy number [12,13].

**Figure 1:**
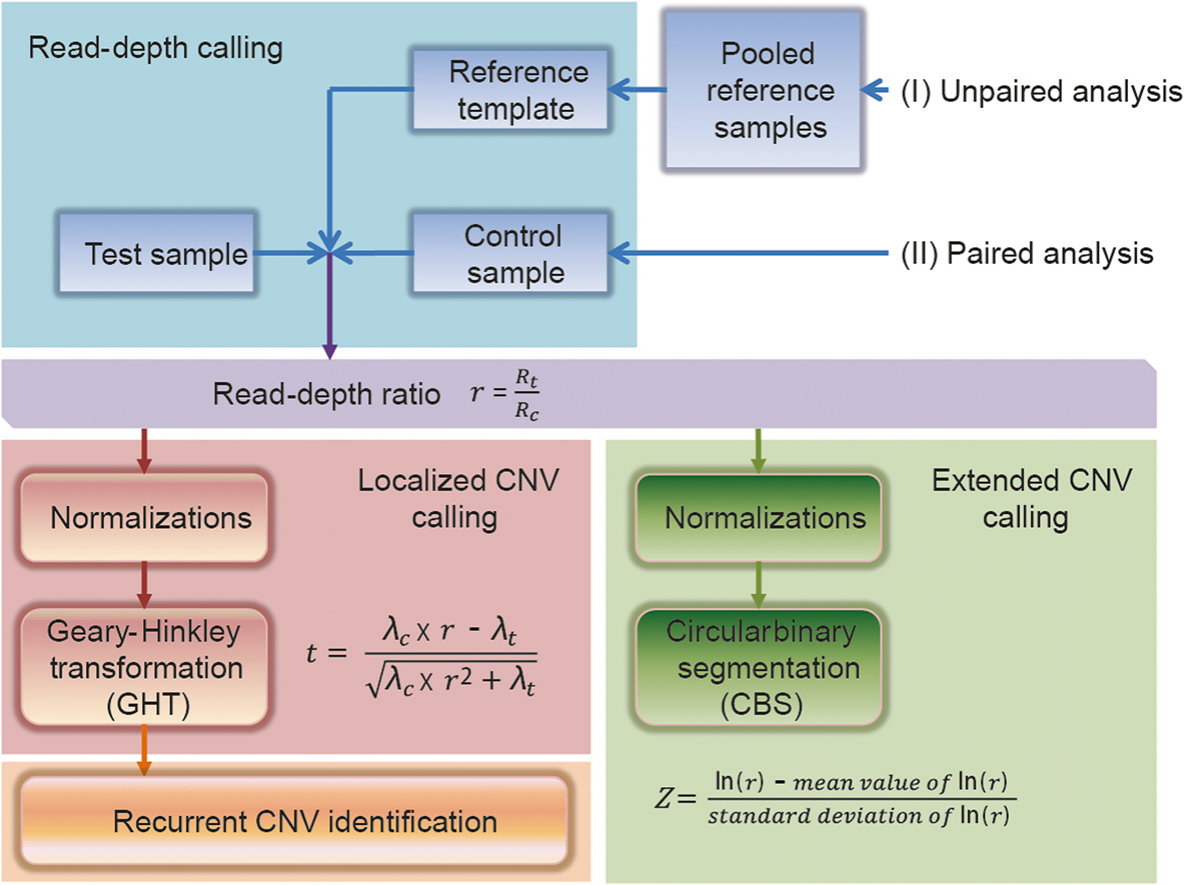
**Schematic diagram of the AluScanCNV calling method.** CNV calling is conducted employing the test sample either with a reference template constructed from pooled reference samples in (I) unpaired analysis, or with a paired control sample in (II) paired analysis, to yield read-depth ratios. GHT is used to call localized CNVs and in turn recurrent CNVs; or alternately CBS is used to call extended CNVs.

In view of this, an AluScanCNV package has been assembled and optimized in the present study for efficient calling of CNVs from the AluScan of a test sample with or without a paired control. In the calling procedure summarized in Figure 1, the human genome is divided into equal-length windows, the size of which can be varied. Read-depth calling is performed in every window of each sample. For paired sample analysis, only those windows with a finite read-depth in both the target and control samples are subjected to CNV calling. For unpaired samples, a reference template is constructed from pooled reference samples by the method of Sathirapongsasuti et al. [3] with adjustment for GC content to enhance robustness, and only those windows with a finite read-depth in both target sample and reference template are subjected to further analysis. CNV calling is performed by two different pathways: (A) Detection of localized CNV is performed using the Geary-Hinkley transformation (GHT) to identify read-depth ratios that could be CNVs. For a group of samples, recurrent CNVs amongst the localized CNVs found are identified based on the assumption that all copy number alterations are independent as invoked in the GISTIC algorithm [14], plus the use of Bonferroni correction; and (B) the circular binary segmentation (CBS) method of Olshen et al. [15] is employed to join together CNV-containing windows with the same copy number into extended CNVs. For both pathways, significant biases due to GC content and total reads are reduced by appropriate normalizations.

In current cancer research, CNV is regarded as an important source of tumorigenesis besides single nucleotide substitution and large structural variation [16,17]. Ovarian cancer, breast carcinoma and lung cell carcinoma for example are categorized as C-class (C stands for CNV) tumors [18], and a variety of cancers are associated with CNVs in tumor suppressor genes and oncogenes such as *TP53* and *RET* [17,19].

Rare constitutional CNVs are well known to be associated with individual cancers, but recurrent constitutional CNVs are usually found to be only low to modest in penetrance suggesting that they could become significant factors in the aggregate [17,20-23]. In our earlier study, recurrent constitutional CNV-features selected by machine learning were found to be capable of distinguishing between genomes with higher predispositions to cancer and those with lower predispositions, and thereby provide a basis for the prediction of generalized cancer predisposition [24]. In the present study, the generality of this approach has been expanded by machine-learning selection of localized as well as recurrent somatic CNV-features with the capability of distinguishing between different types of cancer such as liver versus non-liver cancers.

## Methods

### DNA samples and AluScan sequencing

Inter-*Alu* PCR amplifications were performed on 0.1 μg of each of the DNA samples in Additional file 1: Table S1 using, except where otherwise indicated, the four *Alu*-based PCR primers AluY278T18 (5’-GAGCGAGACTCCGTCTCA-3’), AluY66H21 (5’-TGGTCTCGATCTCCTGACCTC-3’), R12A/267 (5’-AGCGAGACTCCG-3’) and L12A/8 (5’-TGAGCCACCGCG-3’) (0.075 μM each), followed by sequencing of the amplicons with the Illumina-Solexa platform and mapping as described [11]. The AluScan sequences of the blood samples from 23 non-cancer subjects (column 3 of Additional file 1: Table S1) were pooled together for the construction of a “23-sample reference template” for unpaired analysis (Figure 1). Descriptions of the various samples are given in Additional file 2: Table S2.

### Correlation of read-depth

The genome in each DNA sample was divided into contiguous windows 5 kb in size. The read-depth for each window was calculated using the genomeCoverageBed program in BEDtools [25]. The read-depths of the highest 5% were adopted as the 95% quantile value for the read-depth distribution for that sample. Read-depths of larger window sizes (100 kb, 300 kb and 500 kb) were generated by merging the read-depth values of 5 kb windows.

### Calling of GHT-based localized CNVs

In the AluScanCNV procedure, detection of a copy-number gain or loss in a test sample relies on comparison of the read-depth of a sequence window on the test sample with that on either a paired control sample in the case of ‘paired analysis’, or a reference template constructed from pooled reference samples in the case of ‘unpaired analysis’, yielding in either case the read-depth ratio for that particular window (Figure 1). The source codes for the AluScanCNV procedure including read-depth calculation are given in Additional file 3: Source code of AluScanCNV.

In calling localized CNVs, the read-depth distribution *R* in any window is assumed to be a Poisson distribution *Po*(*λ*) with parameter *λ*:

1 $$ R\sim Po\left(\lambda \right) $$

which fits *R* into *Po*(*λ*) with *λ* representing the mean value of the distribution. Since the sums of Poisson-distributed random variables will belong to a Poisson distribution if each of those independent random variables is Poisson-distributed, it follows that:

$$ \begin{array}{c}\hfill {R}_1\sim Po\left({\lambda}_1\right)\hfill \\ {}\hfill {R}_2\sim Po\left({\lambda}_2\right)\hfill \\ {}\hfill {R}_3\sim Po\left({\lambda}_3\right)\hfill \end{array} $$

*R*_*n*_ ~ *Po*(*λ*_*n*_) are independent, and therefore

2 $$ {R}_c=\left({\sum}_{i=1}^n{R}_i\right) \sim Po\left({\lambda}_c\right) $$

where

$$ {\lambda}_c={\lambda}_1+{\lambda}_2+{\lambda}_3+\dots +{\lambda}_n={\sum}_{i=1}^n{\lambda}_i $$

Hence a reference template can be constructed by grouping together a series of reference samples for calculating the read-depth ratio of a corresponding window on an unpaired test sample.

The Poisson distribution in Eqn. 1 can be approximated by a normal distribution if the average read-depth in the window is sufficiently high to yield [4]:

3 $$ R\sim N\left(\mu,\ {\sigma}^2\right) $$

Since mean value *μ* and variance *σ*^*2*^ are equal in a normal distribution, both can be represented by *λ*:

4 $$ R\sim N\left(\lambda,\ \lambda \right) $$

For a test sample:

5 $$ R\sim N\left({\lambda}_t,\ {\lambda}_t\right) $$

where *λ*_*t*_ represents the mean read-depth value of all the windows analyzed in the test sample. For a reference template or paired control:

6 $$ R\sim N\left({\lambda}_c,\ {\lambda}_c\right) $$

where *λ*_*c*_ represents the mean read-depth value of all the windows analyzed in a control sample in the case of paired analysis, or in a reference template in the case of unpaired analysis. With either unpaired or paired analysis, only those windows that display a finite read-depth in the test sample as well as a finite read-depth in the reference template or paired control are analyzed.

The read-depth ratio $z$ between test sample and reference template or paired control at the same window is given by:

7 $$ z=\frac{R_t}{R_c} $$

where *R*_*t*_ represents the read-depth value of a given window in test sample, and *R*_*c*_ represents that of the corresponding window in reference template or paired control. Upon adjustment for total reads, we have:

8 $$ r=z\times \frac{N_c}{N_t} $$

where *N*_*t*_ = Σ *R*_*t*_ and *N*_*c*_ = Σ *R*_*c*._ The distribution of *r* is complex. However, when both *R*_*t*_ and *R*_*c*_ are normally distributed, under certain conditions the distribution of $r$ can be approximately transformed into variable *t* using the GHT, or Geary-Hinkley transformation [26].

9 $$ t = \frac{\uplambda_c\times r-{\uplambda}_t}{\sqrt{\uplambda_c\times {r}^2+{\uplambda}_t}} $$

where λ_*t*_, λ_*c*_ and *r* are respectively given by Eqn. 5, Eqn. 6, Eqn. 8.

**Figure 2:**
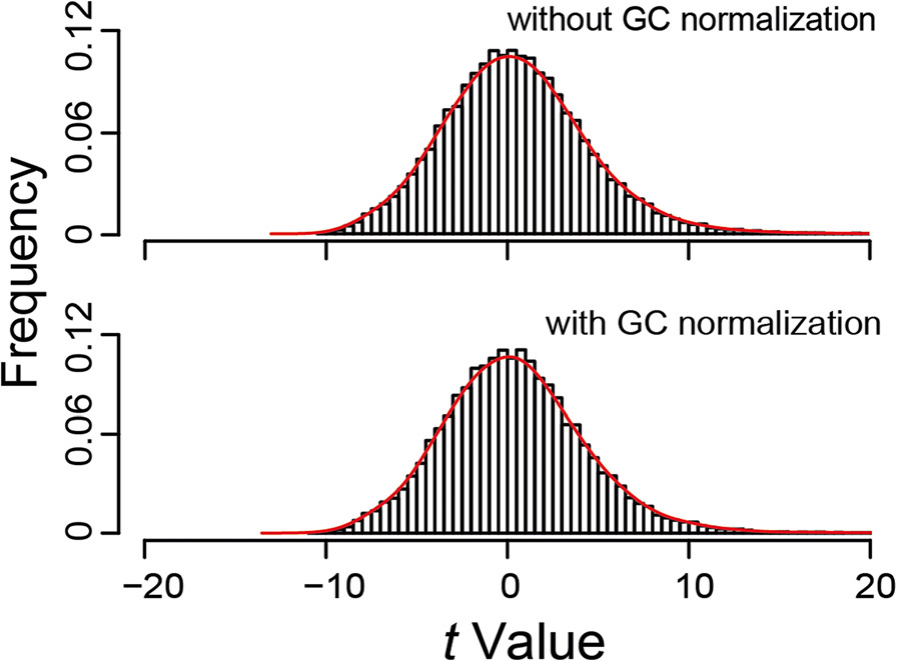
**Distribution of transformed** ***t***-**values.** Upper panel - without GC content normalization; and lower panel - with GC content normalization. Y-axis shows the frequency and X-axis shows the *t*-value from Eqn. 9 or 10. The *t*-values were estimated from the AluScan of GL2B as test sample compared with the 23-sample reference template, and window size was 5 kb.

To normalize with respect to GC content, the windows in a genome are divided into 20 groups based on GC content levels with a 5% increment from one level to the next, and Eqn. 9 becomes:

10 $$ {t}^{\hbox{'}} = \frac{\uplambda_c^{\hbox{'}}\times r-{\uplambda}_t^{\hbox{'}}}{\sqrt{\uplambda_c^{\hbox{'}}\times {r}^2+{\uplambda}_t^{\hbox{'}}}} $$

where ${\uplambda}_t^{\hbox{'}}$ represents the mean value of read-depths in all the windows in a GC-content group in the test sample, and ${\uplambda}_c^{\hbox{'}}$ that in the same GC-content group in the reference template or paired control; *r* is again given by Eqn. 8. Both the distributions of transformed *t*-values (5 kb window size) based on Eqn. 9 and Eqn. 10 fit the standard normal distribution (Figure 2).

For variable *t* in Eqn. 9, its cumulative standard normal distribution function is *Φ*(*t*), and we have:

11 $$ p = 2\ \left(1 - \varPhi (t)\right)\kern1.75em  if > 1 $$

12 $$ p = 2\ \varPhi (t)\kern0.75em  if $$

Copy-number gain is called for a window when *p* <0.05 and $r>$1, and copy-number loss is called for a window when *p* <0.05 and $r<$ 1. No CNV is called for a window if *p*$\ge$0.05 or r =1. *Φ*(*t*) in Eqn. 11 and Eqn. 12 is replaced by *Φ*(${t}^{\prime }$) when Eqn. 10 is used instead of Eqn. 9.

Since the GHT represents a key step in CNV calling using Eqns. 11 and 12, a CNV called using these equations may be referred to as a GHT-based localized CNV in distinction from CNVs that are called by other means.

According to Chiang et al. [27], the theoretical minimum window size for CNV detection is determined by the required power, sequencing amount, coverage size and reference genome size. In the present study, AluScans with ~30 M reads covering ~150 M unique sequences (Additional file 2: Table S2) were aligned to the ~3 Gb human genome. On this basis, 50 kb would be the theoretical minimum window size for power >0.99, which however has to be increased for higher accuracy in CNV calling [4].

### Identification of recurrent CNVs

After the GHT-based localized CNVs have been detected in a group of samples using a reference template or paired control (Figure 1), matrix *M* is constructed as follows with each row representing a window, and each column representing a sample. All the samples must show a finite number of reads in a given window for that window to be included in the matrix *M*:

$$ M = \left[\begin{array}{cc}\hfill 1\hfill & \hfill 0\hfill \\ {}\hfill 0\hfill & \hfill 1\hfill \\ {}\hfill 0\hfill & \hfill 1\hfill \end{array}\right] $$

Thus *M* is an “m $\times$ n” matrix with m candidate windows (rows) and n samples (columns). Each element in *M* takes on a binary value of 0 or 1, with 1 representing ‘CNV identified’ and 0 representing ‘no CNV identified’. *M*_*ij*_ therefore describes the CNV status of the *i*th window in the *j*th sample. *Mi*∙ stands for the CNV status at window *i* across all samples; and *M*∙*j* stands for the CNV status at all the windows in sample *j*.

**Figure 3:**
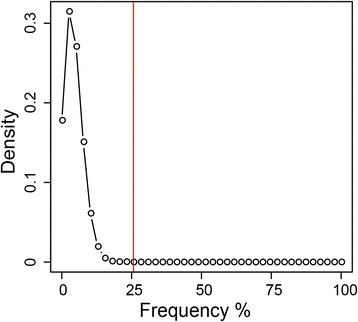
**Poisson binomial distribution of CNVs among samples.** The frequency for any window is the percentage of total samples that display a CNV at that window, and the density is the fraction of all the windows analyzed that display a given frequency. Accordingly, CNVs that give rise to frequencies to the right of the cut-off frequency (indicated by red line) represent CNVs that occur at an exceptionally high percentage of samples with *p* <0.01, and are therefore regarded as recurrent CNVs. The curve shown was calculated using localized CNVs called from the AluScans of the 38 cancer samples in column 2 of Additional file 1: Table S1, in each case employing for comparison the 23-sample reference template.

Based on the assumption that all copy number alterations are independent [14], *P*(*k*) the distribution of CNVs in the different samples is described by the Poisson binomial distribution of a sum of independent Bernoulli trials [28]:

13 $$ P(k)={\displaystyle \sum_{A\in {F}_k}}{\displaystyle \prod_{\alpha \in A}}{p}_{\alpha }{\displaystyle \prod_{\beta \in {A}^c}}\left(1-{p}_{\beta}\right) $$

where *F*_*k*_ is the set of all subsets of *k* integers encountered, *A* the set of matrix elements with value ‘1’, *A*^*C*^ the set of matrix elements with value ‘0’, ${p}_{\alpha }$ the frequency of ‘1’ elements in the samples and ${p}_{\beta }$is the frequency of ‘0’ elements in the samples. Based on Eqn. 13, the ‘poibin’ package in R-program [29] is employed to calculate the cut-off frequency in the *P*(*k*) distribution that gives rise to *p* <0.01, which is the criterion for the identification of a recurrent CNV (Figure 3).

### Calling of CBS-based extended CNVs

To identify extended CNVs that cover multiple windows, the CBS, or circular binary segmentation algorithm [15] is employed to join together neighboring windows with the same read-depth ratio into an extended CNV segment. In this instance, GC content normalization is performed using the following equation [30]:

14 $$ {D}_{corrected}={D}_{global} \cdot \times \cdot {D}_{raw}/{D}_{GC} $$

where *D*_*global*_ represents the median read-depth across the genome of a test sample, a reference sample or a paired control, *D*_*raw*_ a read-depth before GC correction, and *D*_*GC*_ the median read-depth for windows in the same GC content group. By obtaining the *D*_*corrected*_ of a test sample, viz. *D*_*t*-*corrected*,_ and that of the corresponding window on the reference template or paired control, viz. *D*_*c*-*corrected*,_ the GC-corrected read-depth ratio is given by:

15 $$ r=\left[{D}_{t- corrected}\right]/\left[{D}_{c- corrected}\right] $$

**Figure 4:**
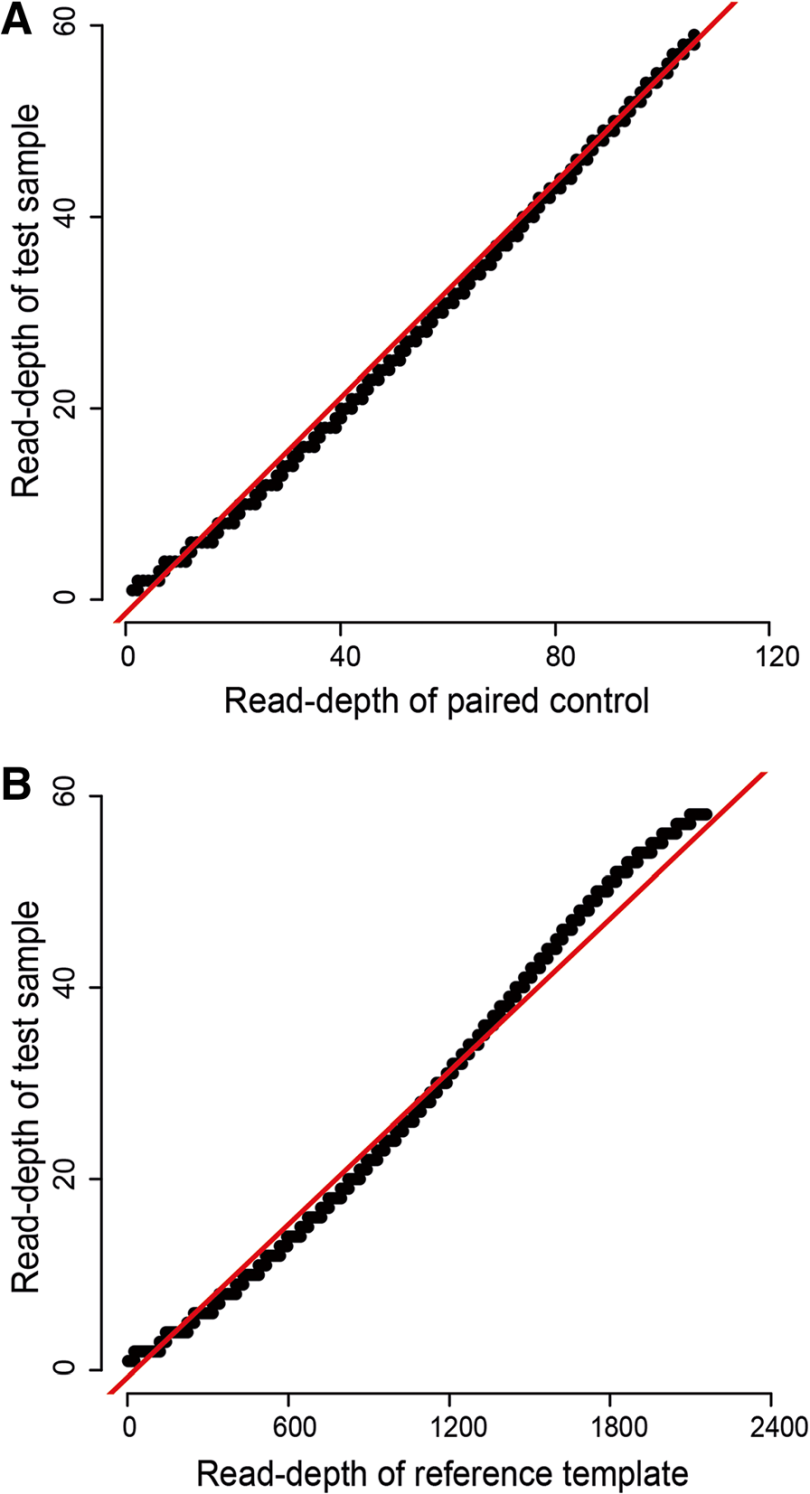
**Q**-**Q plot of read**-**depth distributions.** In paired analysis **(A)**, test glioma tissue GL2T was compared to its paired non-cancerous control GL2B; Pearson’s correlation coefficient was 0.9986. In unpaired analysis **(B)**, the same test sample was compared to the 23-sample reference template; Pearson’s correlation coefficient was 0.9939. The read-depths of 5 kb windows are represented by densely overlapping solid circles, and the red lines are the linear regression lines.

Thereupon r is converted into a *Z* score by means of Eqn. 16 prior to application of the CBS algorithm:

16 $$ Z=\frac{ \ln (r) - mean\  value\  of\  \ln (r)}{standard\  deviation\  of\  \ln (r)} $$

where the ‘mean value of ln(*r*)’ and ‘standard deviation of ln(*r*)’ refer to the ln(*r*) values across all the analyzed windows of the test sample. On this basis, a significant difference between the *Z* scores of any two neighboring windows displaying a copy number gain or copy number loss will indicate a discontinuity that rules out the possibility of the two neighboring windows belonging to the same extended CNV. Otherwise, without such discontinuity, these neighboring CNVs will be regarded as part of an extended CNV. Notably, a high correlation between the read-depth distributions of test sample and that of reference template or paired control is a prerequisite to CBS-based CNV calling. The quantile-quantile (Q-Q) plots in Figure 4 show that such a high correlation in fact prevailed for the AluScan sequence data obtained under the experimental conditions described in the Methods section for paired analysis using a paired control as well as unpaired analysis using a reference template.

The source codes implementing modified cnv.R in CNV-seq [4] and DNAcopy [31] in R-program for CBS-based calling of extended CNVs are included in Additional file 3: Source code of AluScanCNV.

### Machine-learning selection of CNV-features to classify different types of cancers

The application of localized or recurrent somatic CNV-features from the collection of CNVs identified from AluScans of cancer samples by means of AluScanCNV to distinguish between different types of cancers was performed as previously described for the use of recurrent constitutional CNV-features to distinguish between constitutional genomes with high versus low cancer-predispositions [24]. Distinguishing CNV-features were selected using the correlation-based feature selection method (CfsSubsetEval) [32,33] with BestFirst search from the Weka package [34], and classification of samples was carried out with 1,000 iterations of two-fold cross validation employing the Naïve Bayes algorithm. Accuracy of classification was evaluated in terms of AUC, viz. ‘Area Under the receiver operating characteristic Curve’ and the F-score given by:

$$ F- score = 2TP/\left(2TP + FP + FN\right) $$

where TP, FP and FN represent true positives, false positives and false negatives respectively.

Clustering of samples was performed with the Euclidean distance method and ward.D cluster method of the ‘pvclust’ package in R [35].

## Results and discussion

The AluScanCNV package depends on two important prerequisites for CNV calling from AluScan sequences. First, there must be a close approximation of the GHT-derived *t*-distribution to a normal distribution in order to call localized CNVs and recurrent CNVs. Secondly, there should be a close correlation between the read-depths in the test sample and paired control or reference template in using CBS to call extended CNVs: while this is not essential for the application of CBS, it provides important extra assurance for the appropriateness and accuracy of such application. While close correlation between test sample and its paired control in this regard might be expected, it needs to be verified that a close correlation exists between test sample and a reference template constructed from reference samples.

In Figure 2, where the AluScans for blood sample GL2B and the 23 non-cancer reference samples that gave rise to the reference template were all performed with four *Alu*-based PCR primers as described in Methods, the *t*-values derived from read-depth ratios through the GHT conformed closely to a normal distribution either with or without GC normalization, thereby confirming the applicability of the GHT to AluScan sequence data. Since the *t*-distribution was well represented by a normal curve even without GC normalization in this example, the contribution made by GC normalization was not manifest. However, the advantage of GC normalization has been pointed out by other workers [7]. Moreover, in Additional file 4: Figure S1, where a mismatch was introduced such that the AluScan for the test sample was conducted using only three *Alu*-based primers, whereas the reference-sample AluScans were carried out using four *Alu*-based primers, the deviation of the *t*-distribution from a normal curve was pronounced without GC normalization, but substantially improved with GC normalization, indicating that GC normalization enhanced the robustness of GHT-based CNV calling.

Q-Q plots in Figure 4A and 4B show that the high correlation between the read-depths of the test sample GL2T and those of its paired control GL2B (4A: Pearson’s coefficient =0.999), and the high correlation between the read-depths of GL2T and those of the reference template (4B: Pearson’s coefficient =0.994). The results therefore confirmed that a close correlation was obtained in both cases, and the use of the CBS algorithm to call extended CNVs from AluScans is valid when AluScan sequencing is performed employing the experimental conditions described in the Methods section.

### Calling of GHT-based localized and recurrent CNVs

**Figure 5:**
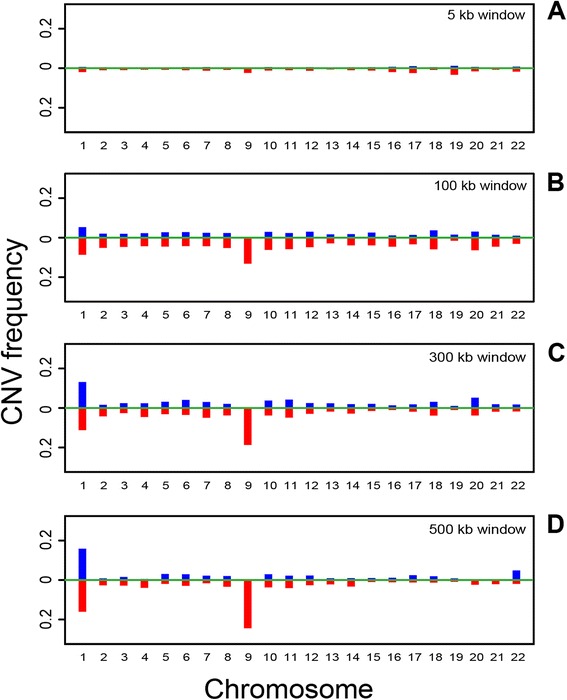
**Chromosomal distribution of localized CNVs called using different window sizes.** GHT-based localized CNVs were called from GL2T AluScan compared to the 23-sample reference template using 5 kb **(A)**, 100 kb **(B)**, 300 kb **(C)** and 500 kb windows **(D)**. CNV Frequency on the y-axis represents the fraction of windows on a chromosome showing CNV gain (upward blue bars) or CNV loss (downward red bars).

**Figure 6:**
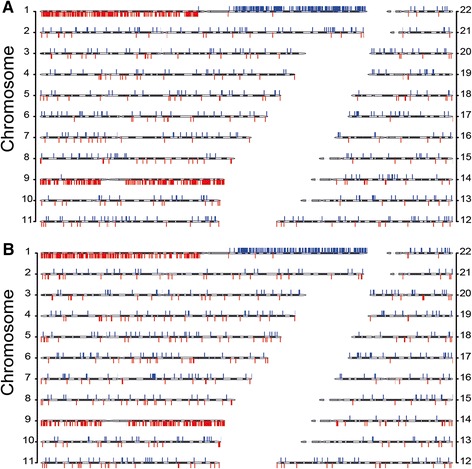
**Chromosomal locations of localized CNVs in a glioma sample using 500 kb windows.** GHT-based localized CNVs were called from AluScan data of glioma tumor tissue GL2T compared to **(A)** its paired blood control GL2B AluScan, and to **(B)** the 23-sample reference template using 500 kb windows. Upward blue bars represent copy number gains, and downward red bars copy number losses.

**Figure 7:**
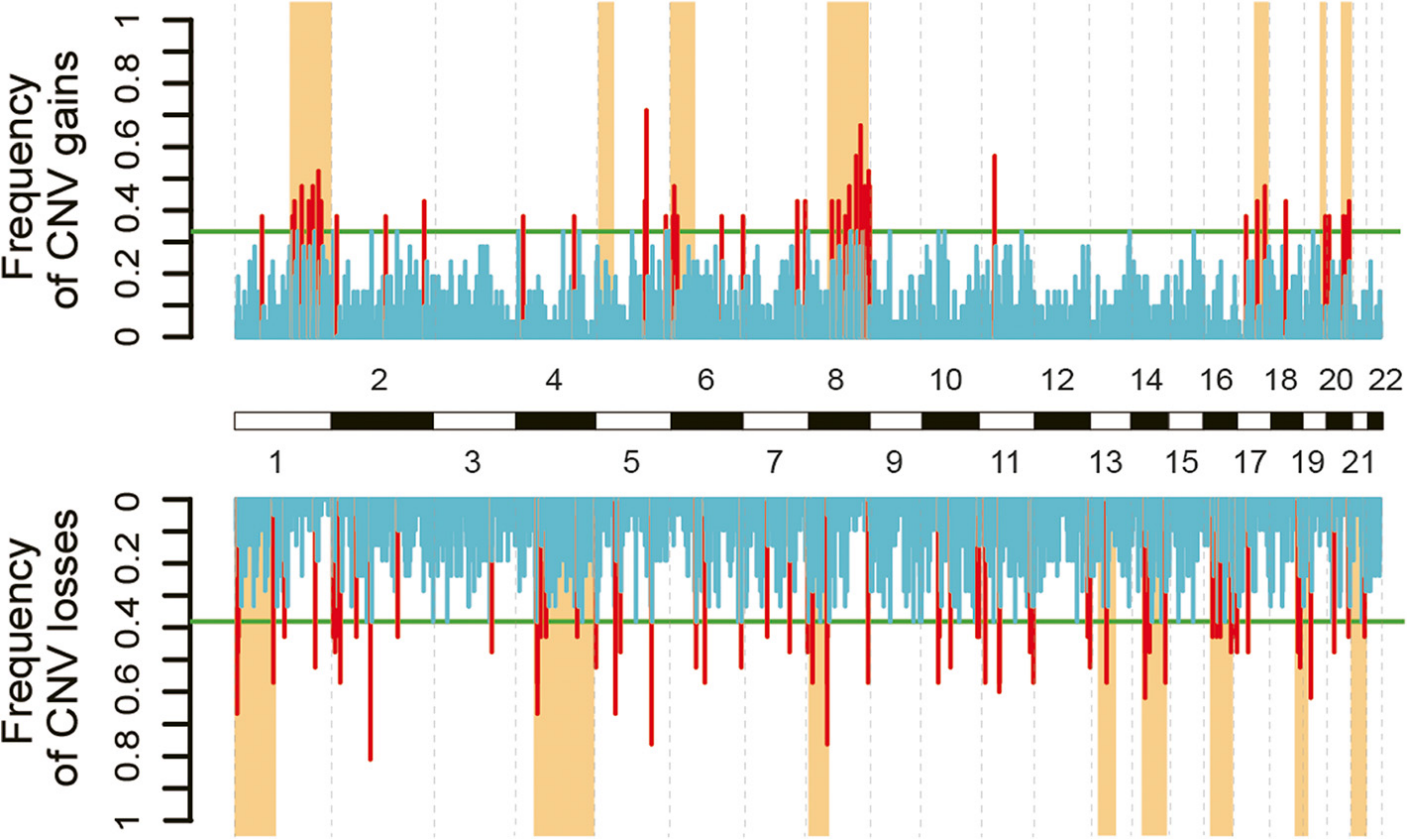
**Chromosomal distribution of recurrent CNVs in twenty**-**one liver cancers.** The 52 recurrent copy number gains (red upward bars) and 99 recurrent copy number losses (red downward bars) were called from the AluScans of 21 liver cancers from Additional file 2: Table S2 using the 23-sample reference template for comparison. Blue bars represent CNVs the frequencies of which did not exceed the green lines marking significant recurrence (*p* <0.01). The orange columns represent CNVs called from WGS data by Kan et al. [36].

**Figure 8:**
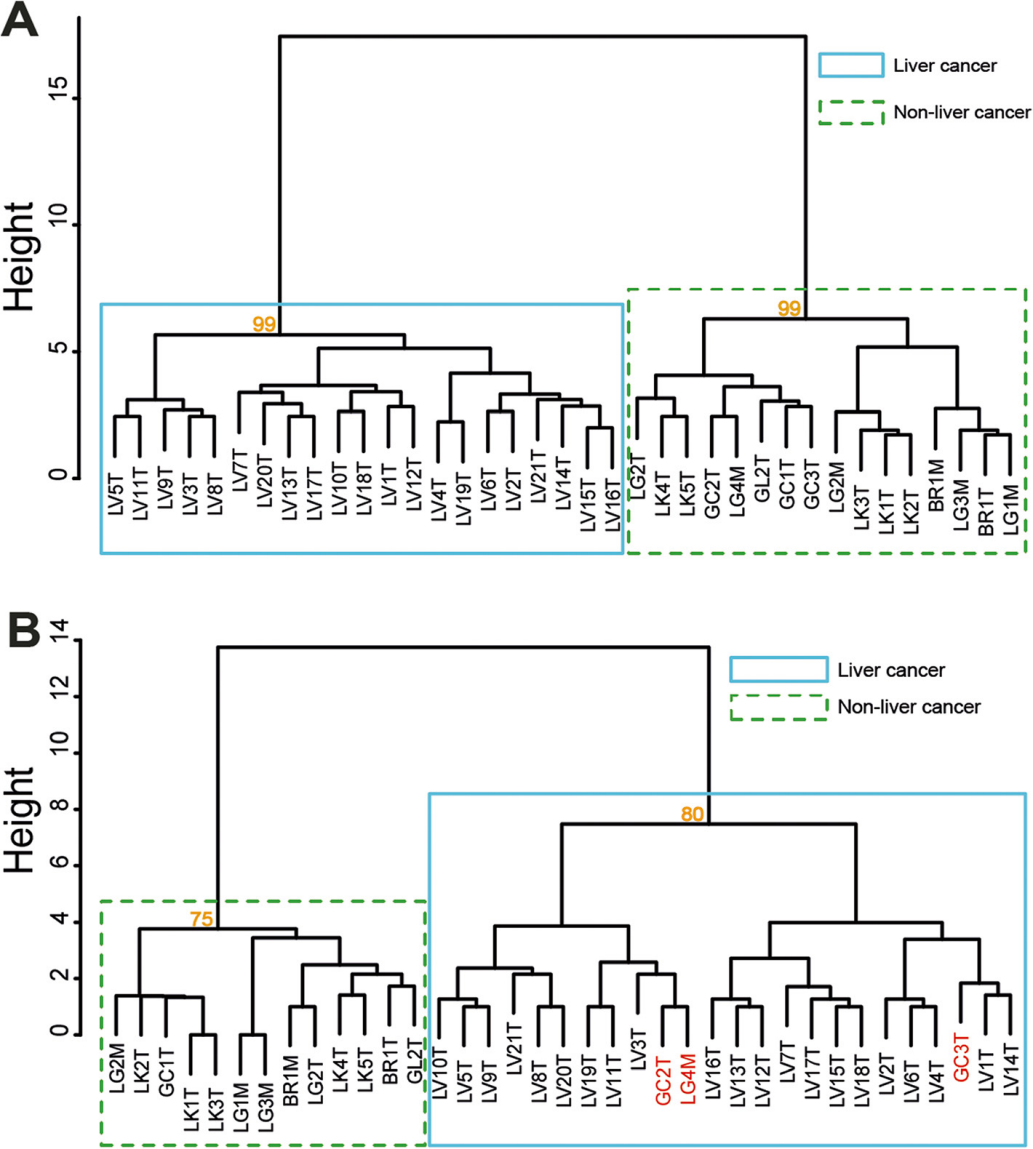
**Hierarchical clusters of liver and non**-**liver cancers based on distinguishing CNV**-**features. (A)** Clustering using localized CNV-features and **(B)** Clustering using recurrent CNV-features. The 21 liver and 16 non-liver cancers analyzed are described in Additional file 2: Table S2. The distinguishing localized and recurrent CNV-features selected by machine learning for the purpose of clustering these two classes of cancers are listed in Additional file 6: Table S3A and 3B respectively. The numbers in orange shown at the nodes for the ‘liver cancer’ (blue solid box) and ‘non-liver cancer’ (green dashed box) clusters indicate the approximate unbiased probabilities, and the three incorrectly clustered samples in Part **(B)** are shown in red. Clustering of samples was performed as described in Methods.

**Figure 9:**
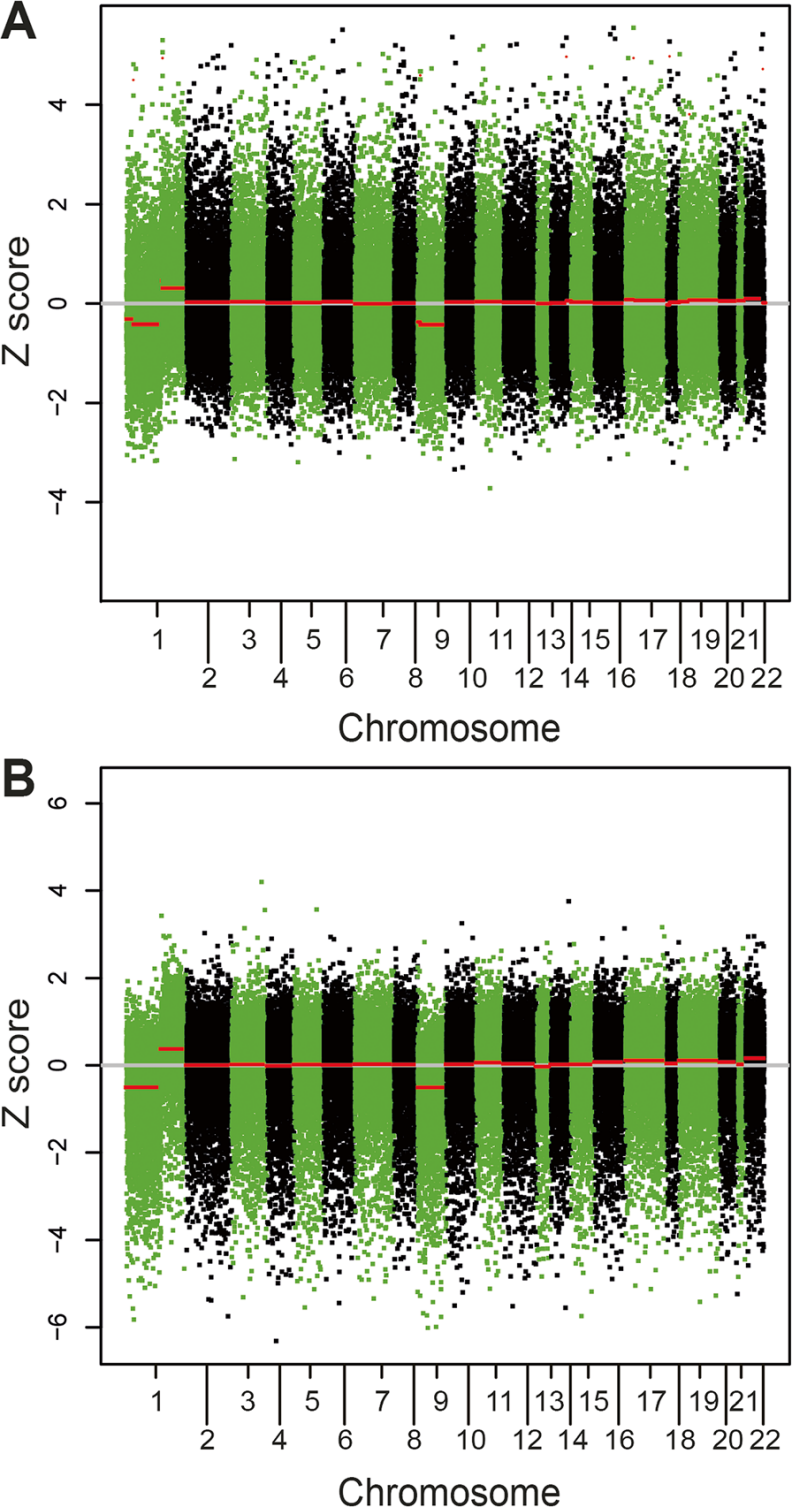
**Chromosomal distribution of extended CNVs in glioma GL2T. (A)** GL2T tumor tissue was compared with either **(A)** paired control blood sample GL2B from the same patient; or **(B)** the 23-sample reference template. The Z scores of windows are shown by green and black dots on alternate autosomal chromosomes. Red horizontal bars with *Z* ≥0.2 represent extended copy number gains, and those with *Z* $\le -$0.2 represent extended copy number losses.

In Figure 5, localized CNVs were called from the AluScan of GL2T tumor cell DNA compared to the reference template employing 5 kb, 100 kb, 300 kb and 500 kb window sizes. The results obtained with all these window sizes indicated that the distribution of CNVs over various autosomal chromosomes were by no means uniform. Instead, they all revealed an enrichment of localized copy number gains in chromosome 1, and enrichment of localized copy number losses in chromosome 1 and 9. The enrichments at these two chromosomes compared to other chromosomes were detectible with the 5 kb and 100 kb windows, and became increasingly prominent with the 300 kb and 500 kb windows. These results were consistent with a decreased impact of sheer chance with the use of larger windows [4,27]. The detailed chromosomal distribution of localized CNVs identified using 500 kb windows further pinpointed the enrichment of copy number losses on chromosomes 1p and 9, and the enrichment of copy number gains on chromosome 1q (Figure 6). In the following analysis, 500 kb windows were employed for localized CNV calling from cancer AluScan sequences (as in Figures 7 and 8), whereas 5 kb windows were employed for extended CNV calling (as in Figure 9).

When recurrent somatic CNVs were called from the AluScans of liver cancers, the distribution of the CNV gains and losses, indicated by red peaks in Figure 7, were unevenly distributed among different chromosomes with a particularly high concentration of CNV gains in chromosomes 1q and 8q, in accord with the CNVs identified from WGS data of liver cancers [36] which are represented by orange column in the figure. This accord between the recurrent CNVs called from AluScans and WGS data provided useful validation for CNV calling from AluScans by means of AluScanCNV.

### Identification of CBS-based extended CNVs

Application of Eqn. 16 to call CBS-based extended CNVs from the AluScan of glioma GL2T yielded Z scores based on a comparison between the test sample and either a paired control (Figure 9A) or the reference template (Figure 9B). Each dot in the plot, colored green and black on alternate autosomal chromosomes 1 to 22 represents the Z score for a window. The CBS-based extended CNVs revealed as red horizontal bars joining up neighboring windows with the same Z score were similar in Figure 9A and 9B, both of which exhibited large extended copy-number losses on chromosomes 1p and 9, and a large copy-number gain on chromosome 1q. The agreement between Figure 9A and 9B confirmed that either a paired control or a reference template can be employed for CNV analysis as indicated in Figure 1. That the extended copy number losses on chromosomes 1p and 9 were both frequently observed in gliomas pointed to the usefulness of AluScanCNV for calling extended CNVs from AluScan sequences.

A comparison between the extended CNV profile of the primary glioma GL1T (Additional file 5: Figure S2) and that of its recurrent cancer GL2T (Figure 9) showed that the two profiles were extensively similar in both paired and unpaired analysis. Therefore cancer recurrence in this instance was not accompanied by any alteration in extended CNVs.

### Cancer classification using machine learning-selected CNV-features

Previously we found that machine learning can be employed to select from microarray-based recurrent CNV-features that are capable of distinguishing between constitutional genomes with a high generalized predisposition to cancer and those with a low predisposition [24]. When this machine learning procedure was applied to the localized or recurrent somatic CNVs called from the AluScans of 21 liver cancers and 16 non-liver cancers shown in Additional file 2: Table S2, 43 localized CNV-features were selected (shown in Additional file 6: Table S3A) for their capability of distinguishing between these two classes of cancers with AUC =1.000 and F-score =1.000 in 1,000 iterations of two-fold cross validation based on the Naïve Bayes algorithm; as shown in the dendrogram in Figure 8A, these localized CNV-features enabled the hierarchical clustering of the 37 cancer samples into the liver and non-liver classes with 100% accuracy. On the other hand, only 12 recurrent CNV-features were selected (shown in Additional file 6: Table S3B) with AUC =0.982 and F-score =0.889 in 1,000 iterations of two-fold cross validation based on the Naïve Bayes algorithm; and these recurrent CNV-features enabled the hierarchical clustering of the 37 cancer samples into the liver and non-liver classes with 34/37 viz. 91.9% internal accuracy, with three incorrect entries as shown in the dendrogram in Figure 8B. It might be noted in this regard that, because the total of 37 cancer samples employed bordered on the minimum for recurrent CNV calling, there is a possibility that the 91.9% internal accuracy attained with the recurrent CNV-features might improve with a larger sample size. The demonstrated internal accuracy clearly showed that the selected CNV-features called by AluScanCNV are highly correlated to cancer-type, and therefore merit in-depth investigation to elucidate the mechanistic basis of such cancer-type correlation. In any event, the findings in Figures 8A and 8B pointed to the utility of CNV calling from AluScan sequences, and the distinguishing power of the machine-selected localized and recurrent CNV-features strongly suggests that such CNV-features are endowed with correlations with cancer types that could lead to valuable insight into type-specific factors underlying the oncogenesis and propagation of different types of cancers.

### Performance on external dataset

**Figure 10:**
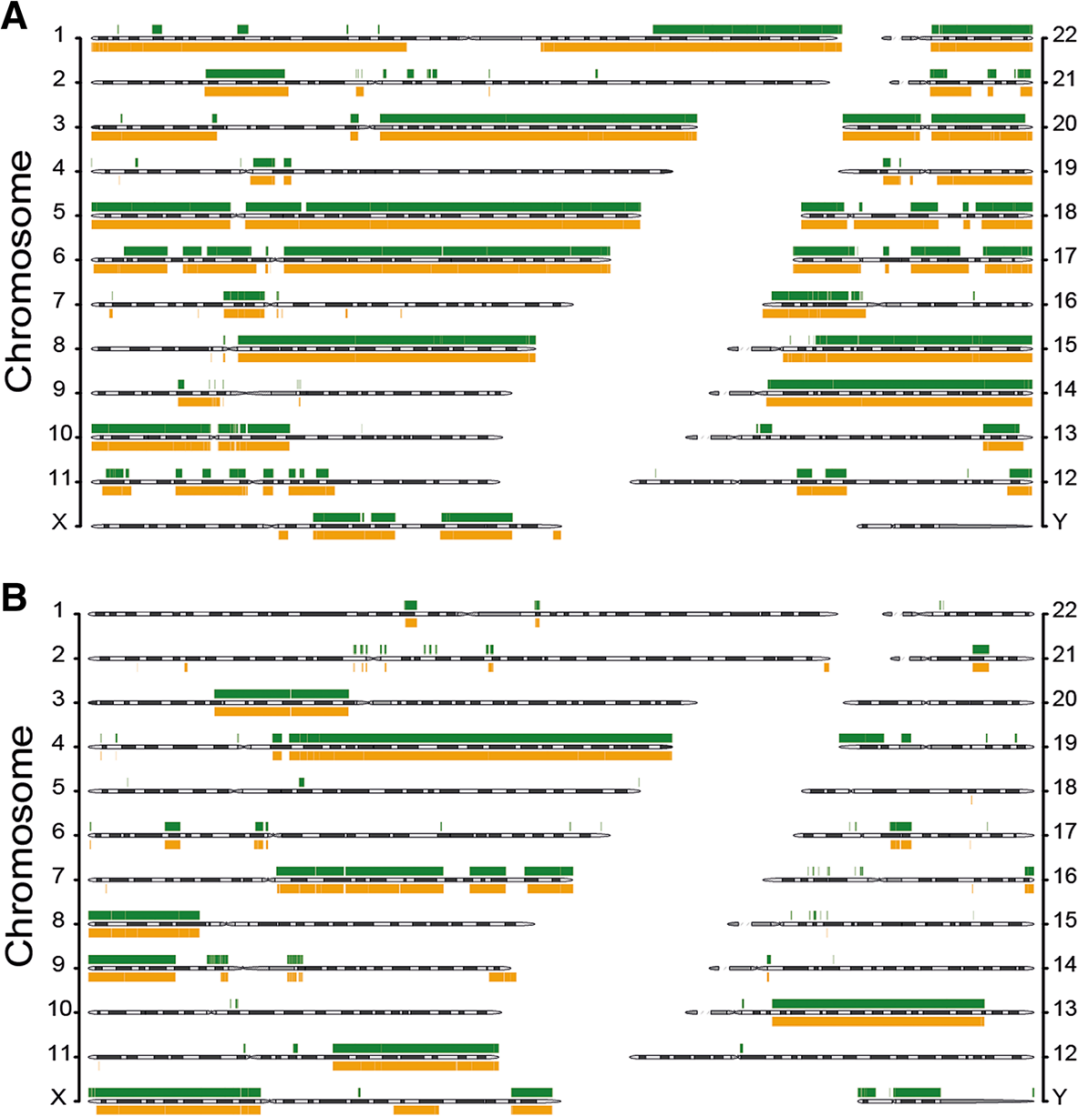
**Comparison of CNV callings by AluScanCNV and FREEC. (A)** Chromosomal distribution of CNV gains obtained by FREEC based on hg18 [7] (green bands above cytobands) or by the CBS-based extended CNV calling in AluScanCNV (orange bands below cytobands). Correlation between the two sets of results yielded Pearson’s R =0.776. **(B)** Chromosomal distribution of CNV losses obtained by FREEC (green bands above cytobands) and by AluScanCNV (orange bands below cytobands). Correlation between the two sets of results yielded Pearson’s R =0.935. The same dataset on cancer cell line HCC1143 from ref.27 was employed in all the CNV estimations. Correlation R values were estimated using the human genome graph function in UCSC (http://genome.ucsc.edu/cgi-bin/hgGenome).

In our results, a two-fold validation was given for the accuracy of our methods. First, the CNVs detected on chromosomes 1 and 9 in glioma GL1T and GL2T have been reported earlier in studies on glioma [37-39]; secondly, the recurrent CNVs on chromosomes 1q and 8q identified in our 21 liver cancers have been reported on a WGS study [36]. As well, to confirm further the accuracy of CNV calling by our methods, external data from a cancer cell line [27] that were used as test data in FREEC [7] were analyzed using our procedure for CBS-based calling of extended CNV. The results obtained were found to be highly correlated with the results obtained with FREEC, yielding Pearson’ R =0.935 in CNV loss calling, and Pearson’s R =0.776 in CNV gain calling (Figure 10).

## Conclusions

The AluScan platform, comprising the usage of inter-*Alu* PCR with multiple *Alu*-based PCR primers to generate a huge range of amplicons for next-generation sequencing, enables the facile capture of *Alu*-proximal sequences that are widespread throughout the human genome. It makes possible a rapid scan of mutations and alterations in diverse genomic regions including exons, introns and other non-coding regions employing only ~0.1 μg DNA samples [11].

The results in Figures 2 and 4 showed that the distribution of *t*-values obtained from AluScan sequences conformed closely to a normal distribution, and the read-depths of a test AluScan sample were closely correlated with those of a paired control AluScan or a reference template constructed from the AluScans of reference samples. These findings established the validity of the AluScanCNV package for calling CNVs from AluScan sequences, which was further confirmed by the properties of the AluScan-derived CNVs identified in various cancer samples.

In Figure 9 and Additional file 5: Figure S2, the large extended copy-number losses identified on chromosomes 1p and 9 in the recurrent GL2T and primary GL1T tumors were entirely consistent with the frequent occurrence of copy number losses at these locations among gliomas [37-39] . Moreover, the localized CNVs of GL2T shown in both panels of Figure 6 clearly pointed to the concentration of localized CNV losses on chromosomes 1p and 9, and concentration of localized CNV gains on chromosome 1q, in complete agreement with the occurrence of extended CNV gains and losses on these chromosomes in Figure 9, even though the calling of localized CNVs and the calling of extended CNVs depend on different approximations: the former requires a close conformation of *t*-values to a normal distribution, whereas the latter requires a close correlation between the read-depths of a test sample and the read-depths of a reference template or paired control.

As well, in Figure 7 the distribution of recurrent somatic CNVs called from AluScans revealed a striking enrichment of CNV gains in chromosomes 1q and 8q compared to other chromosomes. Such enrichment in 1q and 8q likewise represented the most outstanding property of CNVs called from a WGS study [36]: therefore there was excellent agreement in this regard between the CNVs called from AluScans and the CNVs called from WGS. Given the small DNA sample requirement and much lighter data-processing task of AluScan relative to WGS, the AluScan platform would provide an expedited means for characterizing the CNV profiles of normal and diseased human genomes even with small amounts of biopsied tissues. Moreover, because the AluScan method amplifies DNA sequences only from the *Alu* element-rich human genome but not from microbial genomes, it is applicable to the analysis of esophageal, stomach, intestinal, pulmonary and wound samplings etc. with little interference from the presence of microbial DNAs.

When the localized or recurrent CNVs obtained from liver and non-liver cancers derived from AluScans were subjected to machine learning-selection, distinguishing localized or recurrent CNV-features could be selected that enabled a highly accurate classification between liver cancers and non-liver cancers (Figure 8). These results corroborated and expanded our earlier finding that recurrent constitutional CNV-features provided a valuable basis for the classification and prediction of high versus low constitutional predisposition to cancer [24]. In so doing, they have substantiated the usefulness of machine-learning selected CNV-features, both recurrent and localized ones, for identifying CNVs in the germ-line or cancer genomes that are correlated with the attributes of predisposition to cancer and cancer typing. An extension of this CNV-feature based approach to identify the role of CNVs important to other cancer attributes such as cancer staging and susceptibility or resistance to different treatment modalities, as well as the CNVs important to other diseases besides cancers likewise merits in-depth investigation.

### Availability of supporting data

The AluScan sequencing data of the 63 samples listed in Additional file 1: Table S1 are available upon request.

## Additional files

Additional file 1: Table S1. Samples employed for CNV calling in this study. Additional file 2: Table S2. Information on the 63 samples included in Additional file 1. Additional file 3: Figure S1-S12. Source code of AluScanCNV. This file contains all the source code for calling CNV and detecting significantly recurrent CNVs as well as the required input file. All the code files are written in R and perl. Additional file 4: Figure S1. Distribution of *t*-values obtained with mismatched AluScans. Upper panel - without GC content normalization; and lower panel - with GC content normalization. In contrast to Figure 2, where the AluScan of GL2B DNA and the AluScans of the 23-sample reference template were obtained employing the same four *Alu*-based PCR primer set described in Methods, a mismatch in primer sets was introduced in the present figure: the AluScan of GL3B test sample DNA was performed using only three of the four *Alu*-based PCR primers described in Methods (with omission of primer L12A/8), thus differing from the four primers employed in the 23-sample reference template. Additional file 5: Figure S2. Chromosomal distribution of extended CNVs in glioma GL1T. (A) GL1T tumor tissue was compared with either (A) paired control blood sample GL2B from the same patient; or (B) the 23-sample reference template. The Z scores of windows are shown by green and black dots on alternate autosomal chromosomes. Red horizontal bars with *Z* ≥0.2 represent extended copy number gains, and those with *Z* ≤−0.2 represent extended copy number losses. Additional file 6: Table S3. A Localized CNV-features selected from autosomal chromosomes for classification between liver and non-liver cancers. B Recurrent CNV-features selected from autosomal chromosomes for classification between liver and non-liver cancers.

## Abbreviations

CNV: Copy Number Variation
SNP: Single Nucleotide Polymorphism
PCR: Polymerase Chain Reaction
WGS: Whole Genome Sequencing
GHT: Geary-Hinkley Transformation
CBS: Circular Binary Segmentation
