# Supplementary material for: Copy number variation analysis based on AluScan sequences
Source: J Clin Bioinforma. 2014 Dec 5;4:15. doi: 10.1186/s13336-014-0015-z (PMC4273479; doi:10.1186/s13336-014-0015-z)
Supplement: Additional file 1: Table S1. — Samples employed for CNV calling in this study. [file 13336_2014_15_MOESM1_ESM.docx]

Additional File 1. Samples employed for CNV calling in this study.

| **Subject type** | **Cancer tissue** | **White blood cells of non-cancer subjects** |
| --- | --- | --- |
| Autism | 0 | 1 |
| Breast cancer | 2 | 0 |
| Diabetes | 0 | 3 |
| Gastric cancer | 3 | 0 |
| Glioma | 2 | 0 |
| Leukemia | 5 | 0 |
| Liver cancer | 21 | 0 |
| Lung cancer | 5 | 0 |
| Normal subjects | 0 | 11 |
| TCE hyper-sensitivity | 0 | 2 |
| Schizophrenia | 0 | 6 |

Descriptions of the samples are given in Additional File 2.
