# Supplementary figures and images for: Copy number variation analysis based on AluScan sequences
Source: J Clin Bioinforma. 2014 Dec 5;4:15. doi: 10.1186/s13336-014-0015-z (PMC4273479; doi:10.1186/s13336-014-0015-z)

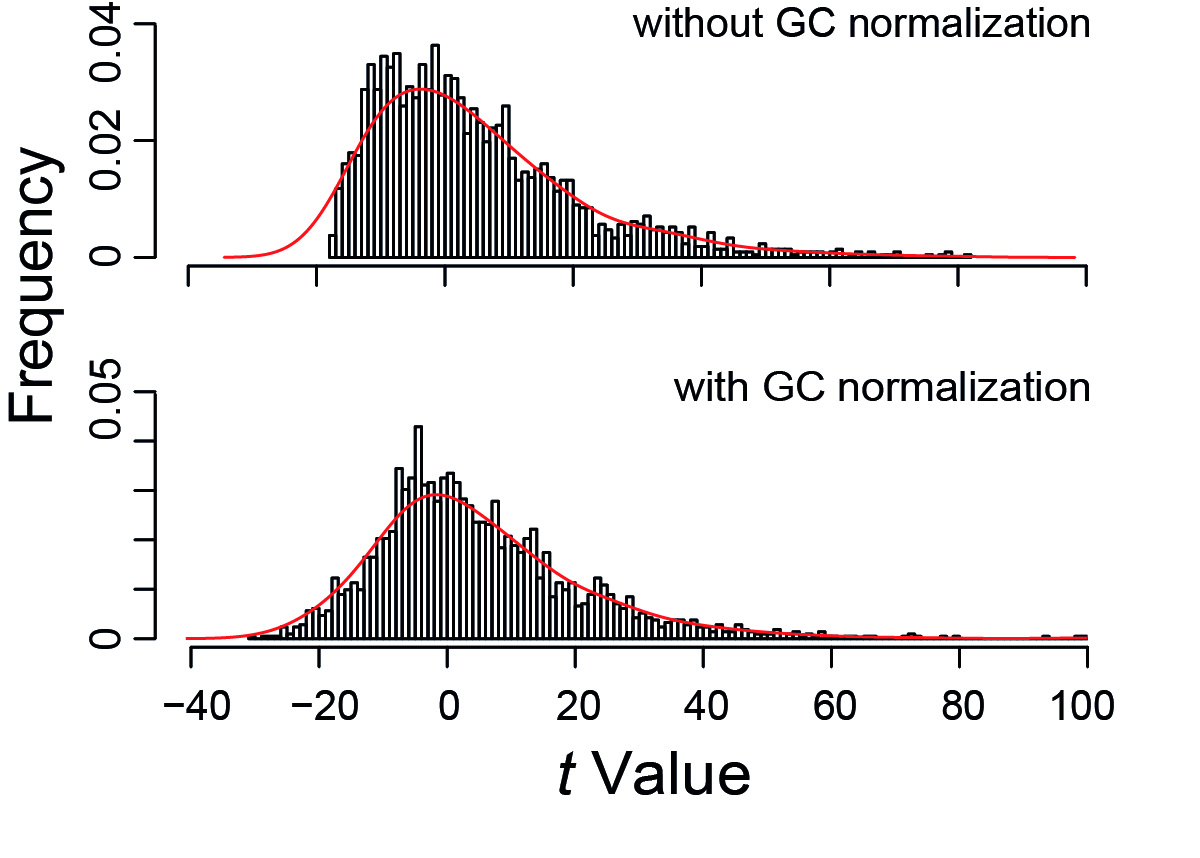

Supplement: Additional file 4: Figure S1. — Distribution of t-values obtained with mismatched AluScans. Upper panel - without GC content normalization; and lower panel - with GC content normalization. In contrast to Figure 2, where the AluScan of GL2B DNA and the AluScans of the 23-sample reference template were obtained employing the same four Alu-based PCR primer set described in Methods, a mismatch in primer sets was introduced in the present figure: the AluScan of GL3B test sample DNA was performed using only three of the four Alu-based PCR primers described in Methods (with omission of primer L12A/8), thus differing from the four primers employed in the 23-sample reference template. [file 13336_2014_15_MOESM4_ESM.tiff]

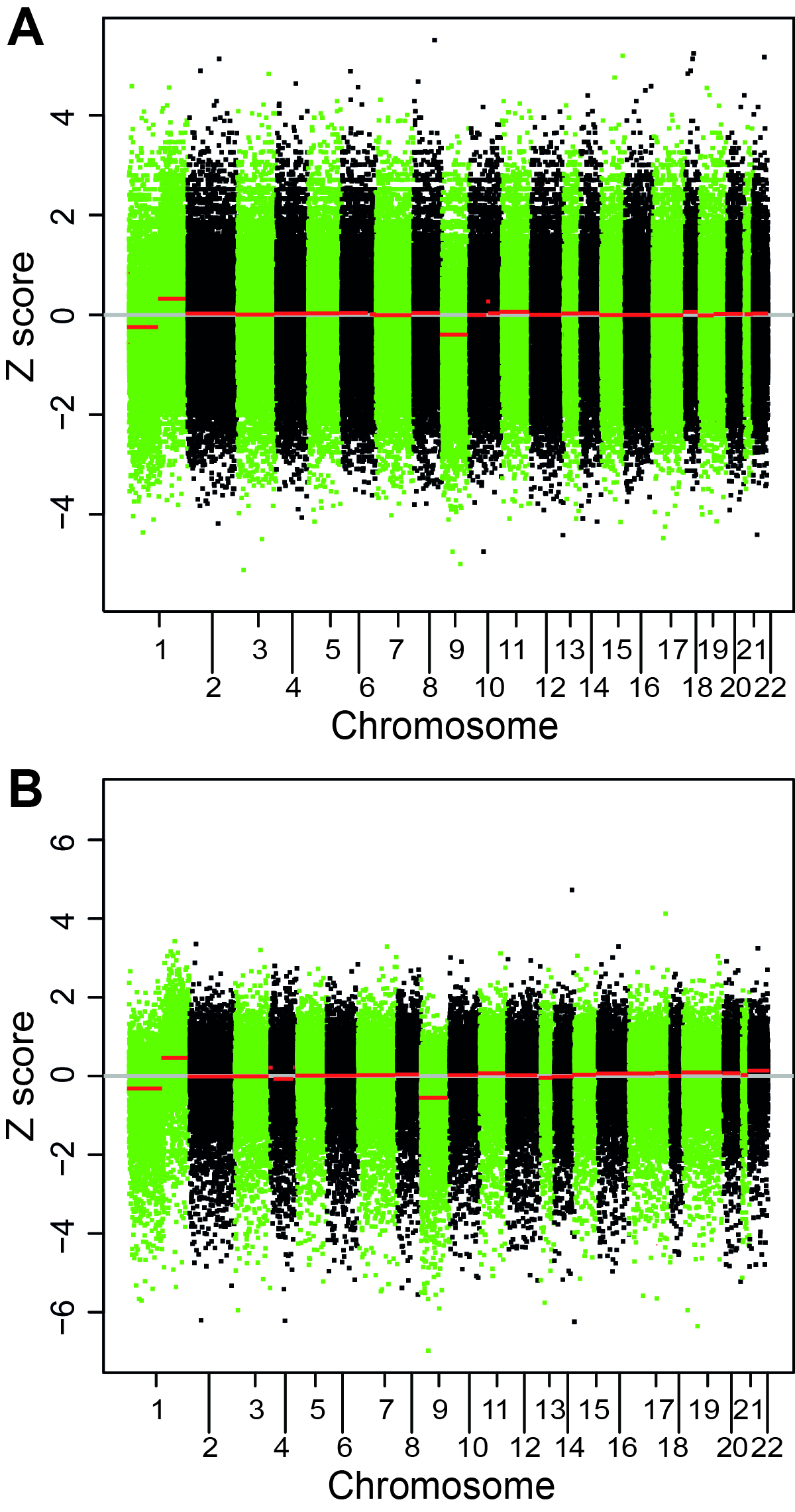

Supplement: Additional file 5: Figure S2. — Chromosomal distribution of extended CNVs in glioma GL1T. (A) GL1T tumor tissue was compared with either (A) paired control blood sample GL2B from the same patient; or (B) the 23-sample reference template. The Z scores of windows are shown by green and black dots on alternate autosomal chromosomes. Red horizontal bars with Z ≥0.2 represent extended copy number gains, and those with Z ≤−0.2 represent extended copy number losses. [file 13336_2014_15_MOESM5_ESM.tiff]
