# Supplementary material for: Copy number variation analysis based on AluScan sequences
Source: J Clin Bioinforma. 2014 Dec 5;4:15. doi: 10.1186/s13336-014-0015-z (PMC4273479; doi:10.1186/s13336-014-0015-z)
Supplement: Additional file 6: Table S3. — A Localized CNV-features selected from autosomal chromosomes for classification between liver and non-liver cancers. B Recurrent CNV-features selected from autosomal chromosomes for classification between liver and non-liver cancers. [file 13336_2014_15_MOESM6_ESM.docx]

Additional File 4A. Localized CNV-features selected from autosomal chromosomes for classification between liver and non-liver cancers

| Number | CNV type | Autosomal chromosome | Start | End |
| --- | --- | --- | --- | --- |
| 1 | Gain | 1 | 31000001 | 31500000 |
| 2 | Gain | 2 | 81000001 | 81500000 |
| 3 | Gain | 3 | 74500001 | 75000000 |
| 4 | Gain | 4 | 74500001 | 75000000 |
| 5 | Gain | 4 | 156500001 | 157000000 |
| 6 | Gain | 4 | 134000001 | 134500000 |
| 7 | Gain | 4 | 97500001 | 98000000 |
| 8 | Gain | 4 | 146500001 | 147000000 |
| 9 | Gain | 5 | 108000001 | 108500000 |
| 10 | Gain | 6 | 84500001 | 85000000 |
| 11 | Gain | 6 | 5500001 | 6000000 |
| 12 | Gain | 7 | 34000001 | 34500000 |
| 13 | Gain | 7 | 108500001 | 109000000 |
| 14 | Gain | 8 | 142500001 | 143000000 |
| 15 | Gain | 8 | 136000001 | 136500000 |
| 16 | Gain | 8 | 140000001 | 140500000 |
| 17 | Gain | 11 | 119500001 | 120000000 |
| 18 | Gain | 12 | 17000001 | 17500000 |
| 19 | Gain | 17 | 71500001 | 72000000 |
| 20 | Gain | 19 | 27500001 | 28000000 |
| 21 | Gain | 20 | 15000001 | 15500000 |
| 22 | Loss | 1 | 29500001 | 30000000 |
| 23 | Loss | 2 | 14500001 | 15000000 |
| 24 | Loss | 3 | 81500001 | 82000000 |
| 25 | Loss | 4 | 167000001 | 167500000 |
| 26 | Loss | 4 | 146500001 | 147000000 |
| 27 | Loss | 5 | 9500001 | 10000000 |
| 28 | Loss | 6 | 92000001 | 92500000 |
| 29 | Loss | 8 | 142500001 | 143000000 |
| 30 | Loss | 8 | 79000001 | 79500000 |
| 31 | Loss | 9 | 42500001 | 43000000 |
| 32 | Loss | 11 | 16500001 | 17000000 |
| 33 | Loss | 11 | 48500001 | 49000000 |
| 34 | Loss | 11 | 11000001 | 11500000 |
| 35 | Loss | 12 | 128000001 | 128500000 |
| 36 | Loss | 12 | 1 | 500000 |
| 37 | Loss | 12 | 106500001 | 107000000 |
| 38 | Loss | 14 | 90500001 | 91000000 |
| 39 | Loss | 15 | 20500001 | 21000000 |
| 40 | Loss | 16 | 55000001 | 55500000 |
| 41 | Loss | 17 | 9500001 | 10000000 |
| 42 | Loss | 18 | 58000001 | 58500000 |
| 43 | Loss | 20 | 59500001 | 60000000 |

Additional File 4B. Recurrent CNV-features selected from autosomal chromosomes for classification between liver and non-liver cancers

| Number | CNV type | Autosomal chromosome | | Start | End |
| --- | --- | --- | --- | --- | --- |
| 1 | Gain | 1 | 220500001 | | 221000000 |
| 2 | Gain | 3 | 148000001 | | 148500000 |
| 3 | Gain | 5 | 121000001 | | 121500000 |
| 4 | Gain | 6 | 120500001 | | 121000000 |
| 5 | Gain | 8 | 124500001 | | 125000000 |
| 6 | Gain | 8 | 143000001 | | 143500000 |
| 7 | Gain | 17 | 71500001 | | 72000000 |
| 8 | Loss | 8 | 142500001 | | 143000000 |
| 9 | Loss | 10 | 71000001 | | 71500000 |
| 10 | Loss | 11 | 51000001 | | 51500000 |
| 11 | Loss | 11 | 48500001 | | 49000000 |
| 12 | Loss | 22 | 16000001 | | 16500000 |
